# A case of *Nocardia asiatica* pneumonia: Relapse caused by trimethoprim-sulfamethoxazole treatment nonadherence

**DOI:** 10.1016/j.idcr.2026.e02606

**Published:** 2026-05-10

**Authors:** Huifen Zuo, Fei Zhao, Chunyan Wu, Jingxin Gu, Na Dong, Xinteng Zhang, Yiming Ying, Ying Guo, Dongyan Shi

**Affiliations:** a Department of Clinical Laboratory, Hebei Yiling Hospital, Shijiazhuang, Hebei Province 050091, China; b The College of Life Science and Food Engineering, Hebei University of Engineering, Handan, Hebei Province 056038, China; c School of Teacher Education, Hebei International Studies University, Shijiazhuang, Hebei Province 050000, China; d Department of Clinical Laboratory of Infectious Disease, The Second Hospital of Hebei Medical University, Shijiazhuang, Hebei Province 050000, China

**Keywords:** Nocardia asiatica, Pulmonary infection, Trimethoprim-sulfamethoxazole, Treatment adherence, Whole-genome sequencing

## Abstract

Nonadherence to trimethoprim-sulfamethoxazole (TMP-SMX) is a known risk factor for nocardiosis treatment failure, often due to adverse effects. However, reports linking relapse to treatment interruption caused by poor adherence are scarce. A 70-year-old male with chronic bronchitis presented with persistent fever, cough, and sputum. *Nocardia asiatica* was identified. Initial improvement followed a 28-day TMP-SMX course, but the patient discontinued therapy. Multiple readmissions over three years resulted from relapses due to poor adherence. Whole-genome sequencing of isolates from the first and third episodes revealed 99.9% genetic similarity, confirming relapse from the original strain. Only after completing a full six-month TMP-SMX course did the patient achieve sustained remission with no recurrence for five years. This case provides molecular evidence supporting that premature TMP-SMX discontinuation contributed to relapse, emphasizing that adherence is as critical as accurate diagnosis for cure.

## Highlights

• After multiple relapses over 3 years, a patient with N. asiatica pneumonia achieved cure with 6-month TMP-SMX.
• WGS confirmed 99.9% genetic identity of serial isolates, ruling out reinfection and showing nonadherence caused relapse.
• Clinical improvement does not equal microbiological cure; adherence monitoring is as critical as diagnostic accuracy.

## Introduction

*Nocardia* species are Gram-positive aerobic actinomycetes responsible for opportunistic infections. The primary risk groups include immunocompromised hosts and individuals with impaired local pulmonary defenses, notably those with underlying structural lung diseases [1], [2], [3]. Pulmonary involvement is the most common clinical manifestation, often posing diagnostic challenges due to its nonspecific symptoms that mimic tuberculosis or conventional pneumonia [4]. The management of nocardiosis is notoriously prolonged, requiring months of continuous antimicrobial therapy to prevent disease relapse. While poor patient adherence to such extended regimens is widely acknowledged as a major barrier to cure, direct microbiological evidence—particularly from advanced molecular methods—correlating nonadherence with relapse is not commonly documented in the literature. We present a case of recurrent pulmonary *N. asiatica* infection in which whole-genome sequencing (WGS) provided strong evidence suggesting that two clinical relapses were associated with premature treatment cessation, highlighting the critical role of adherence in achieving microbiological cure. While the clinical association between poor adherence and relapse is well recognized, direct WGS-based confirmation—ruling out reinfection—has rarely been reported for nocardiosis, and to our knowledge, this is the first such case for *Nocardia asiatica*.

## Case presentation

A 70-year-old retired teacher from Hebei, China, with a history of chronic bronchitis over three decades and prior cholecystectomy, was admitted in August 2018 for a six-month history of intermittent fever, cough, and sputum production following cold exposure. Despite multiple empirical antibiotic regimens—including sequential courses of cefuroxime, levofloxacin, and azithromycin—he experienced only transient improvement followed by recurrent symptoms.

**Fig. 1:**
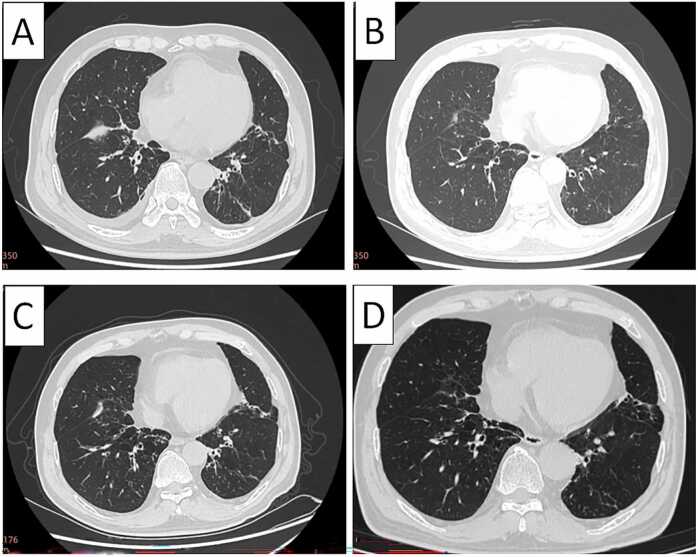
Serial chest CT images. (**A**) Initial presentation (August 2018): Findings at initial presentation. (B) One week post-discharge: Improvement after initial treatment. (C) Third admission: New nodule and effusion during relapse. (D) After completing 6-month therapy: Significant resolution after completed therapy.

Auscultation revealed thick breath sounds with scattered crackles. Chest CT (Fig. 1A) showed chronic bronchitis with emphysema, bilateral bronchiectasis with mild lower-lobe inflammation, and small right-sided pleural effusions. Pulmonary function tests demonstrated a forced expiratory volume in 1 s (FEV1) of 80% of the predicted value and an FEV1/FVC ratio of 72% (normal).

The laboratory findings included: white blood cell count (WBC) 10.16 × 10^9^/L; neutrophil percentage (NEUT%) 72.2%; erythrocyte sedimentation rate (ESR) 23 mm/h; C-reactive protein (CRP) 53 mg/L; and procalcitonin (PCT) 0.25 ng/ml.

The preliminary diagnoses were pneumonia, bronchiectasis, and chronic bronchitis.

Sputum smear revealed gram-positive rods exhibiting uneven staining, filamentous morphology, and characteristic 90-degree branching. A weakly positive acid-fast stain was suggestive of *Nocardia* species. Subsequent culture on blood and TM agar under aerobic conditions at 35°C for 3 days yielded small (1–2 mm), white, dry, smooth colonies. The isolate was ultimately identified as *Nocardia asiatica* by 16S rRNA gene sequencing.

Following admission, the patient was initiated on an empirical antimicrobial regimen ceftazidime and moxifloxacin. After *Nocardia* was confirmed, TMP-SMX (160/800 mg twice daily, approximately 4.9 mg/kg/day of the trimethoprim component based on the patient's weight of 65 kg) was added for 14 days. Follow-up showed normalized inflammatory markers (WBC, ESR, CRP) and significant symptomatic improvement. The patient was discharged on a six-month course of oral TMP-SMX with instructions for regular follow-up. One week post-discharge, a repeat chest CT (Fig. 1B) showed improvement in bronchiectasis and pulmonary inflammation, resolution of the right pleural effusion, and lung re-expansion. The patient discontinued TMP-SMX two weeks post-discharge due to symptomatic improvement. Adherence was assessed through patient interviews and review of clinical records. He reported feeling completely recovered and believed further treatment was unnecessary; he denied any significant adverse effects such as nausea, rash, or gastrointestinal discomfort.

Approximately 10 months after initial discharge, he was readmitted with hemoptysis. He had briefly self-administered TMP-SMX at home, likely explaining the negative cultures despite positive smear for *Nocardia*. He improved after two weeks of inpatient TMP-SMX therapy and continued treatment for approximately one month after discharge, but then discontinued therapy on his own.

A third admission occurred approximately 17 months after the initial presentation. Chest CT (Fig. 1C) demonstrated a new small nodule (≈0.8 cm) in the right lower lobe and a small pericardial effusion. Sputum smear and culture confirmed *N. asiatica*. Whole-genome sequencing (WGS) was performed to clarify whether this represented a relapse or a new infection. The WGS procedures, including DNA extraction, sequencing, and bioinformatic analysis, were performed as previously described by Yang et al. [5]. The sequence data were deposited in the NCBI database under GenBank accession nos. JBSXWC000000000 and JBSVVB000000000. The two isolates exhibited 99.9% genetic similarity, confirming relapse due to incomplete eradication. Drug susceptibility testing was performed on isolates from both the first and third episodes using broth microdilution following CLSI M24S-Ed2 guidelines. Both isolates showed identical susceptibility profiles. The isolates were susceptible to trimethoprim-sulfamethoxazole (MIC 0.5/9.5 mg/L), linezolid (MIC 0.25 mg/L), imipenem (MIC ≤1 mg/L), amikacin (MIC 1 mg/L), ceftriaxone (MIC 0.5 mg/L), minocycline (MIC 1 mg/L), tobramycin (MIC ≤1 mg/L), and clarithromycin (MIC 1 mg/L), and resistant to ciprofloxacin (MIC >4 mg/L), moxifloxacin (MIC >8 mg/L), and amoxicillin-clavulanate (MIC 64/32 mg/L).

The patient was treated with TMP-SMX (oral, 160/800 mg twice daily) and ceftriaxone (intravenous, 2 g once daily) for 8 days with clinical improvement. On this occasion, he subsequently adhered to a full 6-month course of oral TMP-SMX. A follow-up chest CT approximately 18 months after the third admission (Fig. 1D), showed significant resolution of the pulmonary nodules, pericardial effusion, and infectious parenchymal changes, along with overall improvement in the background of chronic bronchitic and emphysematous features. Sputum smear and culture were negative on two separate occasions.

The prescribed and actual treatment durations for each episode were as follows: first episode, prescribed 6 months, actual 28 days (patient discontinued voluntarily); second episode, prescribed 6 months, actual approximately 6 weeks (patient stopped after improvement); third episode, prescribed 6 months, actual 6 months (completed). Adverse effects were denied by the patient as a reason for discontinuation in all episodes.

No relapse has occurred since, with follow-up through 2025 (five years after treatment completion).

**Fig. 2:**
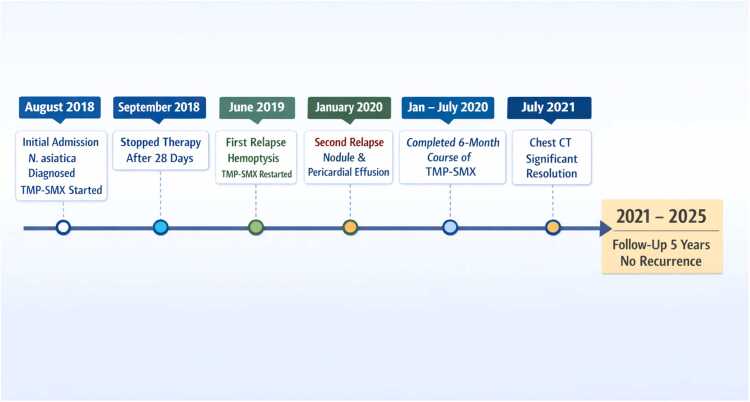
Timeline of clinical events. Key events include initial diagnosis (August 2018), treatment discontinuation after 28 days (September 2018), first relapse with hemoptysis (June 2019), second relapse with new pulmonary nodule and pericardial effusion (January 2020), completion of 6-month TMP-SMX course (January–July 2020), radiographic improvement (July 2021), and 5-year follow-up without recurrence (through 2025).

A comprehensive timeline of the patient's clinical course is presented in Fig. 2.

## Discussion

Nocardiosis, an uncommon but life-threatening opportunistic infection caused by aerobic *Nocardia* species, presents with a broad clinical spectrum. While pulmonary, cutaneous, and central nervous system involvements predominate [6], [7], the pathogen can also cause rare but serious infections at other sites, including infective endocarditis, intra-abdominal and iliopsoas abscesses, osteomyelitis, keratitis, and retinitis [8], [9], [10], [11], [12]. This diagnostic process, stemming from its varied and nonspecific presentation, is clearly reflected in our patient.

In the present case, the patient’s six-month history of recurrent fever, cough, and sputum initially led to misdiagnosis as bacterial pneumonia and ineffective empirical antibiotic therapy with ceftazidime and moxifloxacin. The definitive diagnosis was achieved after sputum microscopy revealed characteristic branching, weakly acid-fast filaments, later confirmed as *Nocardia asiatica* by culture and 16S rRNA sequencing. His longstanding chronic bronchitis and bronchiectasis likely created a susceptible environment by compromising local pulmonary defenses, underscoring the need to consider nocardiosis in patients with structural lung disease who do not respond to therapy.

Nocardiosis management is tailored to disease severity and infection site. TMP-SMX remains the cornerstone of therapy for mild cases, while severe or disseminated infections often require combination regimens such as carbapenems, amikacin, or linezolid [13]. Extended treatment duration—typically 6 months for localized pulmonary disease and ≥ 12 months for disseminated or CNS infection—is commonly used to prevent relapse, though the optimal duration of therapy is uncertain [13]. This case strongly suggests that treatment courses shorter than six weeks are inadequate, but it does not provide evidence on whether durations between six weeks and six months might be sufficient.

The patient improved after 2–4 weeks of TMP-SMX but self-discontinued it. Multiple readmissions over three years resulted from nocardiosis relapse. Whole-genome sequencing (WGS) was performed on isolates from the first and third episodes to distinguish relapse from reinfection. Sequencing was conducted on an Illumina NovaSeq platform with > 100 × coverage, following methods previously described by Yang et al. [5]. The two isolates differed by only 2 single nucleotide polymorphisms (SNPs), which is well below the commonly accepted threshold of < 20 SNPs for defining the same bacterial strain. This high genetic identity (99.9%) confirms that the third episode was a relapse caused by the original strain, rather than a reinfection with a new environmental strain. However, we acknowledge that environmental isolates were not sequenced, and reinfection with a genetically similar strain cannot be completely excluded.

We acknowledge that while WGS confirmed the same strain, causality between nonadherence and relapse cannot be definitively established from a single case. Several alternative explanations should be considered. First, the initial treatment duration (28 days) might have been insufficient for microbiological eradication, even with full adherence. However, the patient's clinical improvement during initial therapy and the subsequent relapses that occurred only after treatment cessation suggest that nonadherence was a major contributing factor. Second, host factors—particularly his long-standing chronic bronchitis and bronchiectasis—likely impaired local pulmonary defenses, increasing his susceptibility to both initial infection and relapse. These structural abnormalities may have created a niche for residual bacteria that could reactivate after treatment withdrawal. Third, regarding host factors, the patient had no known immunosuppressive conditions (e.g., HIV, diabetes mellitus, malignancy) and was not receiving any immunosuppressive therapy (e.g., corticosteroids, chemotherapy, biologics) at the time of initial presentation or during follow-up. Incomplete bacterial clearance in this case may also be attributed to disease severity and host pharmacokinetic factors. The patient's underlying structural lung disease likely created a biofilm-friendly environment, allowing residual bacteria to persist despite adequate serum drug concentrations. Additionally, while TMP-SMX penetration into pulmonary tissue and secretions is generally good, individual variations in drug absorption or metabolism cannot be completely ruled out without therapeutic drug monitoring, which was not performed. Fourth, while we cannot completely rule out the possibility that a shorter course (e.g., 3–4 months) might have been sufficient, this case clearly demonstrates that a course shorter than six weeks is inadequate. The optimal duration for localized pulmonary nocardiosis in patients with structural lung disease remains to be determined. Despite these limitations, the temporal association between treatment cessation and relapse, the WGS confirmation of the same strain, and the ultimate cure after completing six months of therapy collectively support nonadherence as the primary cause of relapse in this patient.

A potential consideration is the distinction between airway colonization and true infection, particularly in a patient with chronic structural lung disease. While *Nocardia* colonization can occur in bronchiectasis, several features in this case support active infection rather than colonization: (i) persistent fever (up to 38.5°C) and productive cough; (ii) new radiological findings including a pulmonary nodule (≈0.8 cm) and pericardial effusion on chest CT during relapse (Fig. 1C); (iii) elevated inflammatory markers (CRP 53 mg/L, ESR 23 mm/h); and (iv) clear clinical and microbiological response to TMP-SMX therapy, with negative follow-up cultures after treatment completion. Furthermore, the relapses occurred specifically after treatment cessation, and WGS confirmed the same strain, making incidental colonization an unlikely explanation for the recurrent symptomatic episodes.

In summary, the patient’s clinical trajectory—characterized by two episodes of treatment discontinuation initiated voluntarily by the patient, subsequent disease relapse, and ultimate microbiological cure achieved only upon full completion of the prescribed antimicrobial course—represents a compelling natural clinical experiment. This clinical course unequivocally illustrates that clinical improvement does not correspond to microbiological eradication. Therefore, successful clinical management necessitates a dual-pronged strategy: diagnostic precision must be integrated with rigorous adherence monitoring. This encompasses not only accurate pathogen identification and optimal antimicrobial agent selection but also the implementation of a robust, proactive follow-up protocol to confirm treatment completion over the extended therapeutic course, thereby ensuring definitive pathogen eradication.

## CRediT authorship contribution statement

**Yiming YING:** Writing – review & editing. **Ying GUO:** Writing – review & editing. **Dongyan SHI:** Writing – review & editing. **Chunyan WU:** Writing – review & editing. **Jingxin GU:** Writing – review & editing. **Na DONG:** Writing – review & editing. **Xinteng ZHANG:** Writing – review & editing. **Zuo Huifen:** Writing – original draft. **Fei ZHAO:** Writing – original draft.

## Author contributions

HZ and FZ were involved in the literature search and drafted the manuscript. DS, CW, JG, ND, XZ, YY and YG made critical revisions to the manuscript. All the authors read and approved the final manuscript.

## Ethics approval

Ethical approval was not required for this case report.

## Consent

Written informed consent was obtained from the patient for publication of this case report and accompanying images. A copy of the written consent is available for review by the Editor-in-Chief of this journal on request.

## Funding

This work was supported by the Hebei Provincial Administration of Traditional Chinese Medicine [Grant No. 2024143] and the Health Commission of Hebei Province [Grant No. 20242248].

## Declaration of Competing Interest

The authors declare the following financial interests/personal relationships which may be considered as potential competing interests: Huifen ZUO reports financial support was provided by Hebei Provincial Administration of Traditional Chinese Medicine. Huifen ZUO reports financial support was provided by Health Commission of Hebei Province. If there are other authors, they declare that they have no known competing financial interests or personal relationships that could have appeared to influence the work reported in this paper.

## Data Availability

The whole-genome sequencing datasets generated during this study have been deposited in the NCBI repository under GenBank accession nos. JBSXWC000000000 and JBSVVB000000000. No other additional data are available.

## References

[1] Lo S.H., Chin C., Lu P.L., Lin S.Y. (2022). Nocardial brain abscess in a patient with AIDS. Int J Infect Dis.

[2] Zhou B., Gong H., Liu S. (2025). Nocardia cyriacigeorgica Infection in a COPD Patient. Clin Lab.

[3] Zuo H., Ye J., Li C., Li S., Gu J., Dong N., Zhao Y., Hao J., Song M., Guo Y., Gao W., Zhao Z., Zhang L. (2024). Myasthenia gravis complicated with pulmonary infection by *Nocardia cyriacigeorgica*: a case report and literature review. Front Med (Lausanne).

[4] Hays W.B., Czosnowski Q. (2022). Continuous etomidate for the management of cushing's syndrome complicated by pulmonary nocardiosis. J Pharm Pract.

[5] Yang J., Jiang T., Zhang M., Xue J., Shi D. (2025). Phenotypic and genotypic profiles of clinical isolates of various *Nocardia* species to carbapenems and fluoroquinolones. J Antimicrob Chemother.

[6] Li T., Chen Y.X., Lin J.J., Lin W.X., Zhang W.Z., Dong H.M. (2022). Successful treatment of disseminated nocardiosis diagnosed by metagenomic next-generation sequencing: a case report and review of literature. World J Clin Cases.

[7] Salazar Reinoso F.R., Romero-Santana G.S., Mawyin Juez A.E., Calderon Navas J.A., Ronquillo Piloso A.S. (2023). Brain abscess due to *Nocardia asiatica* in a 63-year-old patient with adult-onset still disease: a case report. Clin Case Rep.

[8] Fayos M., Severo A., García-Cosío M.D., Prados C., Alonso M., López-Medrano F. (2024). *Nocardia* and mucoral co-infection in heart transplant recipient. Rev Esp Quim.

[9] Gurnani B., Moshirfar M. (2025). Nocardia Keratitis, StatPearls [Internet].

[10] Yetmar Z.A., Khodadadi R.B., Chesdachai S., McHugh J.W., Challener D.W., Wengenack N.L., Bosch W., Seville M.T., Beam E. (2023). Mortality after nocardiosis: risk factors and evaluation of disseminated infection. Open Forum Infect Dis.

[11] Maraki S., Mavromanolaki V.E., Detorakis E.E., Stafylaki D., Moraitis P., Scoulica E. (2022). *Nocardia elegans* primary iliopsoas abscess: a case report and literature review. Acta Microbiol Immunol Hung.

[12] Ziogou A., Giannakodimos A., Giannakodimos I., Baliou S., Tsantes A.G., Ioannou P. (2025). *Nocardia* osteomyelitis in humans-a narrative review of reported cases, microbiology, and management. Pathogens.

[13] Yetmar Z.A., Marty P.K., Clement J., Miranda C., Wengenack N.L., Beam E. (2025). State-of-the-art review: modern approach to nocardiosis-diagnosis, management, and uncertainties. Clin Infect Dis.

